# TB tracer teams in South Africa: knowledge, practices and challenges of tracing TB patients to improve adherence

**DOI:** 10.1186/1471-2458-13-801

**Published:** 2013-09-04

**Authors:** Claire C Bristow, Laura Jean Podewils, Liza Ellen Bronner, Nonkqubela Bantubani, Martie van der Walt, Annatjie Peters, David Mametja

**Affiliations:** 1 Global AIDS Program South Africa, Centers for Disease Control and Prevention (CDC), Pretoria, South Africa; 2 Division of Tuberculosis Elimination, Centers for Disease Control and Prevention(CDC), 1600 Clifton Road NE, MS E-10, Atlanta, Georgia 30333, USA; 3 South African Medical Research Council (MRC), Western Cape, South Africa; 4 Republic of South Africa National Department of Health, Tuberculosis Unit, Pretoria, South Africa

**Keywords:** Tuberculosis, Default tracing, Treatment management, KAP, Treatment adherence, Community outreach

## Abstract

**Background:**

In 2008–2009 the South African National Tuberculosis (TB) Program (NTP) implemented a national pilot project, the TB Tracer Project, aiming to decrease default rates and improve patient outcomes. The current study aimed to inform the NTP by describing the knowledge, attitudes, and practices of TB program personnel involved with tracing activities.

**Methods:**

A self-administered written questionnaire was sent to TB staff, managers and tracer team leaders to assess basic TB knowledge, attitudes and practices. Descriptive statistics were used to summarize results and the chi-squared statistic was used to compare responses of staff at facilities that participated in the TB Tracer Project (tracer) and those that followed standard NTP care (non-tracer).

**Results:**

Of 560 total questionnaires distributed, 270 were completed and returned (response rate 48%). Total TB knowledge ranged from 70.8-86.3% correct across all response groups. However, just over half (range 50–59.3%) of each respondent group was able to correctly identify the four components of a DOT encounter. A patient no longer feeling sick was cited by 72.1% of respondents as the reason patients fail to adhere to treatment. Tracer teams were viewed as an effective means to get patients to return to treatment by 96.3% of health facility level respondents. Tracer team leaders reported concerns including lack of logistical support (41.7%), insufficient physical safety precautions (41.7%), and inadequate protection from contracting TB (39.1%). Upon patients returning to treatment at the clinic, facilities included in the TB Tracer Project were significantly more likely to discuss alternate DOTS arrangements than non-tracer facilities (79.2 vs. 66.4%, p = 0.03).

**Conclusions:**

This study identified key components of knowledge, attitudes, and practices regarding TB patient tracing activities in South Africa. Educating patients on the essential need to complete treatment irrespective of clinical symptoms may help improve treatment adherence. Future scale-up and integration of TB tracing activities as part of standard TB management should include provisions for standardized training of personnel on the critical elements of DOTS, and for ensuring appropriate supervision, logistical support, and physical safety and TB transmission protection of tracing teams.

## Background

Tuberculosis (TB) is a curable disease, yet 8.8 million persons worldwide are newly infected and 1.45 million persons die each year from TB [1]. Adherence to a standard course of treatment is paramount to achieving an effective cure of TB disease. Failure to cure persons with infectious TB disease promotes drug-resistant strains, increases patient risk for morbidity and mortality, and facilitates continued community transmission [2-5]. However, despite widespread adoption of the directly-observed therapy short-course (DOTS) strategy for over a decade, treatment adherence and default from treatment continue to be a primary challenge in TB control.

Home visits and community outreach are often employed as methods to optimize treatment adherence among TB patients. Previous research has consistently demonstrated the effectiveness of such patient tracing activities in returning patients to care and improving final treatment outcomes [6-11]. However, the ability to conduct these activities may be constrained by limitations in human and logistic resources, particularly in resource-limited settings with a high TB burden.

In South Africa, where the TB burden is considered the 3rd highest in the world [1], DOTS coverage is reported to be 100% [12]; yet, treatment success rates remain below global targets and the burden of drug-resistant TB strains continues to escalate [1].

In 2008–2009, the South African National TB Program (NTP) implemented a national pilot project, the TB Tracer Project, aiming to decrease default rates and improve patient outcomes, by creating teams dedicated to tracing TB patients [13]. The intervention has been described previously, but in brief, teams of health care workers were employed at health facilities to trace TB patients who had interrupted treatment or had missed scheduled follow up appointments for monitoring sputum status [14]. The project was implemented as a NTP programmatic intervention, therefore tracer team activities varied by sub-district. A recent evaluation utilizing data from routine national TB surveillance demonstrated that sub-districts that participated in the project (tracer) had significantly greater decreases in patient default rates and significantly greater increases in treatment success rates over the project period, compared to sub-districts that were not part of the project (non-tracer) (default rate decline 2.8% tracer vs. 0.7% non-tracer; success rate increase 2.6% tracer vs. 0.8% non-tracer)[13,14]. However, further examination is needed to better understand activities utilized for tracing patients and identify reasons that may help explain the differences between tracer and non-tracer sites.

The current study aimed to describe the knowledge, attitudes, and practices of health care and TB program personnel involved with tracing activities and to evaluate the differences between health facilities that participated in the TB Tracer Project (tracer) and health facilities that provided standard NTP patient services during the Project period (non-tracer).

## Methods

### Study population

A survey was conducted in 2010 to include all districts that had participated in the TB Tracer Project (2008–2009). In brief, districts were selected for the Project by provincial and national TB program managers based on designation as having a high burden of TB disease and/or high rates of treatment default; the TB managers at each district selected sub-districts for inclusion in the Tracer Project. There were 21 district and 65 sub-districts that participated in the TB Tracer Project. Within each sub-district, a convenience sample of health facilities was selected to participate.

For the current evaluation, self-administered questionnaires were distributed to five respondent groups involved in TB control at the district and sub-district level: (1) district TB program managers, (2) sub-district TB program managers, (3) tracer facility TB managers, (4) non-tracer facility TB managers, and (5) tracer team leaders. A 30% random sample of health facilities that participated in the project (tracer) and 30% random sample of health facilities in the same sub-districts that did not participate in the project (non-tracer) were also selected. The questionnaire was sent to the TB manager at each selected facility. The tracer team leaders (n = 72) were also sent questionnaires for completion. Questionnaires for the survey were distributed and collected by TB personnel in each province. Questionnaires were sent via mail to the tracer team leaders with stamped return envelopes.

### Questionnaires

Standardized questionnaires were sent to each provincial TB office to distribute to the participating district and sub-district offices and health facilities. Individuals that were in a position to answer the questions (e.g., TB program manager) and had been working during the TB Tracer Project were asked to participate. The self-administered questionnaires aimed to assess basic TB knowledge, attitudes, and practices of persons involved with TB management and tracing TB patients. Basic demographic information was collected from each respondent, including age, gender, education, number of years working in TB, and TB-related training. The questionnaires included a core set of questions across all participant groups, but were tailored to address differing roles of each group.

#### Knowledge

A set of sixteen questions were used to assess basic TB knowledge, including TB etiology, transmission, clinical signs and symptoms, risk factors, diagnosis, treatment and monitoring, and infection [15]. Questions were multiple choice, with the majority involving a single correct answer. However, some of the questions required multiple answers (e.g., identifying all first line TB medications) to be considered correct.

#### Attitudes and perceptions

Questionnaires included items aiming to better understand general perceptions about the usefulness of tracing activities and key reasons for not routinely conducting tracing activities as part of standard TB management. Participants were also asked open-ended questions to provide their views on the primary reasons that TB patients failed to adhere to TB treatment. Tracer team leaders were asked specific multiple-choice and open-ended questions about their experience during the project.

#### Practices

Participants were asked about program practices related to the provision of TB treatment to patients, including number of health care staff, clinic operating hours, DOTS models available to patients, and approaches used to trace patients.

### Ethics

This evaluation was reviewed and approved by the Institutional Review Boards of the US and South African Centers for Disease Control and Prevention and the South African Medical Research Council. Written, informed consent was obtained from all participants who completed and returned the questionnaire.

### Analysis

Descriptive statistics were used to summarize general TB knowledge among each of the groups and for the total sample. A TB knowledge summary score was calculated by dividing the sum of the number of correct responses by the total number of questions and multiplying by 100 to reflect a percentage. Knowledge summary scores were compared between tracer and non-tracer TB health facility managers.

Attitudes, perceptions, overall acceptability, and practices of the TB Tracer Project were summarized using descriptive statistics overall and for each of the five respondent groups: district TB Managers, sub-district TB managers, health facility TB managers (both from tracer and non-tracer sites), and TB Tracer team leaders. For characteristics and questions that were asked to both tracer and non-tracer facility participants, comparisons were conducted using chi-squared test and Student’s t-test. Significance was defined at a p-value of less than or equal to 0.05. All analyses were conducted using Stata 11.0 (College Station, Texas).

## Results

**Figure 1:**
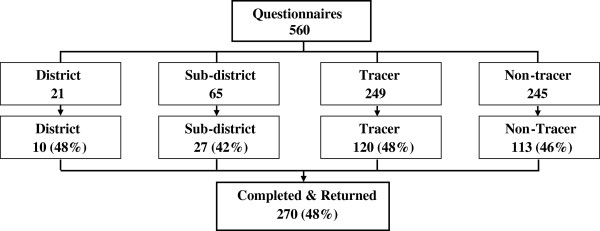
**Distribution and response rates among TB managers at health facilities and program offices included in the study.** *25 of 72 Tracer team leaders returned completed questionnaires.

Of 560 total questionnaires distributed, 270 were completed and returned (response rate 48%; Figure 1). Of the 270 returned, 10 were from TB managers at the district TB office, 27 were from sub-district TB personnel, 120 from tracer TB managers and 113 from non-tracer TB managers. Response rates were similar among tracer and non-tracer TB managers at health facilities (48 vs. 46%, respectively). An additional 25 questionnaires were returned from individual TB tracer team leaders, yielding a 35% response rate (25/72). A total of 295 questionnaires were available for the current study analysis.

### Respondent and program characteristics

Overall, the average age of questionnaire respondents was 47.2 years (SD 10.2);, tracer team leaders tended to be older (mean 63.6 years, SD 6.5) than other respondent groups (Table 1). Most respondents (92.2%) were female, had a tertiary education at a college or university, and were currently or previously employed as nurses in the health facilities during the TB Tracer Project. Across all response groups, the mean number of years working with TB was 9.8 (SD 8.17). The majority had received clinical training on TB disease (83.1%; 245/295) and 57.0% had attended training on DOTS (168/295).

**Table 1:** Characteristics of respondents to TB tracer project questionnaires (n = 295)

	**District (n = 10)**	**Sub-district (n = 27)**	**Tracer (n = 120)**	**Non-tracer (n = 113)**	**Team Lead (n = 25)**	**Total (n = 295)**
	**n (%)**	**n (%)**	**n (%)**	**n (%)**	**n (%)**	**n(%)**
Age, years mean (SD)	47.9 (SD 6.8)	44.5 (SD 8.1)	46.0 (SD 9.3)	45.4 (SD 9.2)	63.6 (SD 6.5)	**47.20 (SD 10.2)**
Gender						
Female	7 (70)	22 (81.5)	113 (94.2)	105 (92.9)	25 (100)	**272 (92.2)**
Male	3 (30)	5 (18.5)	7 (5.8)	8 (7.1)	0	**23 (7.8)**
Highest level of education						
Primary	0	0	1 (.8)	0	0	**1 (.3)**
Secondary	0	2 (7.4)	12 (10)	15 (13.4)	10 (40)	**39 (13.3)**
College	3 (30)	12 (44.4)	68 (56.7)	59 (52.7)	7 (28)	**149 (50.7)**
University	7 (70)	13 (48.2)	39 (32.5)	38 (33.9)	8 (32)	**105 (35.7)**
Professional Occupation						
Physician	0	0	0	0	0	**0**
Nurse	10 (100)	27 (100)	117 (97.5)	108 (95.6)	5 (20)	**267 (90.5)**
Community Health Worker	0	0	3 (2.5)	4 (3.5)	1 (4)	**8 (2.7)**
Retired Nurse	0	0	0	0	19 (76)	**19 (6.4)**
Number of years working with TB patients, mean (SD)	14.7 (SD 8.3)	9.3 (SD 6.0)	9.6 (SD 7.8)	8.9 (7.6)	13.2 (12.4)	**9.8 (SD 8.2)**
Training Course						
Clinical training on TB disease	10 (100)	26 (96.3)	100 (83.3)	88 (77.9)	21 (84)	**245 (83.1)**
Training on reading Chest x-rays	3 (30)	3 (11.1)	4 (3.3)	3 (2.7)	4 (16)	**17 (5.8)**
Training on DOTS	9 (90)	19 (70.4)	65 (54.2)	60 (53.1)	15 (60)	**168 (57.0)**
Training on sputum microscopy	6 (60)	16 (59.3)	34 (28.3)	29 (25.7)	12 (48)	**97 (32.9)**
Other trainings	5 (50)	10 (37.0)	19 (15.8)	20 (17.7)	7 (28)	**61 (20.7)**

Percents reflect proportion that answered the question.

*SD* standard deviation.

Values represent number and percentage unless otherwise indicated.

The health facilities included in this analysis reported an average of 5.1 persons (SD 9.5) designated for the care and treatment of TB patients at the time of the Tracer Project. On average, health facilities were available 5.3 days (SD 1.0) per week to diagnose and manage TB patients (Table 2). Almost all (94.4%) health facilities offered community based DOTS (219/232) and reported being open for DOTS and clinic visits for an average of 9.4 hours (SD 3.4) per day. The number of days available to provide DOTS to patients each week was slightly but significantly greater on average among tracer sites compared to non-tracer sites [5.5 (SD 0.86) vs. 5.2 (SD 1.2), p = 0.04].

**Table 2:** Attributes of tracer and non-tracer health facilities related to the provision of TB care and treatment

	**Total Mean (SD)**	**Tracer Mean (SD)**	**Non-tracer, Mean (SD)**	p-value*
Persons designated for the care and treatment of TB patients	5.1 (9.5)	5.8 (11.8)	4.4 (16.2)	0.26
Days per week the clinic open to provide TB diagnosis and clinic visits	5.3 (1.0)	5.5 (0.87)	5.1 (1.2)	0.12
Days per week the clinic open to provide DOTS to TB patients	5.3 (1.1)	5.5 (0.86)	5.2 (1.2)	0.04
Hours of operation per day the clinic open to provide DOTS to TB patients	9.4 (3.4)	8.9 (3.4)	10.0 (5.5)	0.08

*SD* standard deviation.

*p-value based on t-test comparison of tracer vs. non-tracer facilities.

### TB Knowledge

Total TB knowledge was similar across all respondent groups, ranging from 70.8-86.3% responses correct (Table 3). Just over half of each respondent group (range 50–59.3%) was able to correctly identify the four components of a DOT encounter.

**Table 3:** Basic TB knowledge among personnel involved with TB management and treatment (n = 295)

	**District (n = 10)**		**Sub-district (n = 27)**		**Team Lead (n = 25)**		**Tracer (n = 120)**		**Non-tracer (n = 113)**		p-value*
	**n**	**(%)**	**n**	**(%)**	**n**	**(%)**	**n**	**(%)**	**n**	**(%)**	
What causes TB?	9	(90)	26	(96.3)	23	(92.0)	111	(92.5)	102	(90.3)	0.54
How is TB spread?	10	(100)	26	(96.3)	24	(96.0)	118	(98.3)	110	(97.3)	0.6
What part of the body is the most common site for TB disease?	9	(90)	27	(100.0)	23	(92.0)	118	(98.3)	102	(90.3)	0.17
What is the strongest known risk factor for the development of TB disease?	9	(90)	20	(74.1)	11	(44.0)	49	(40.8)	61	(54.0)	0.05
What are the symptoms of pulmonary TB disease?	6	(60)	12	(44.4)	15	(60.0)	59	(49.2)	53	(46.9)	0.73
How is active TB disease diagnosed?	9	(90)	27	(100.0)	25	(100.0)	116	(96.7)	108	(95.6)	0.67
If a person with HIV has a negative sputum smear, what laboratory technique is particularly useful to determine whether a patient with HIV has TB?	10	(100)	24	(88.9)	18	(72.0)	96	(80.0)	95	(84.1)	0.42
What is the standard duration of treatment for TB that is drug-susceptible?	10	(100)	27	(100.0)	23	(92.0)	115	(95.8)	106	(93.8)	0.48
Which four drugs are recommended for the initial treatment of TB disease?	10	(100)	26	(96.3)	20	(80.0)	102	(85.0)	99	(87.6)	0.56
If someone with active TB stops taking their medicines before they have completed treatment, what are the potential consequences?	10	(50)	11	(40.7)	9	(36.0)	50	(41.7)	47	(41.6)	0.99
What is DOTS?	10	(90)	26	(96.3)	23	(92.0)	112	(93.3)	107	(94.7)	0.66
What are four components of DOT encounter?	10	(50)	16	(59.3)	13	(52.0)	65	(54.2)	58	(51.3)	0.66
Which TB patients should be given DOTS?	10	(100)	26	(96.3)	21	(84.0)	115	(95.8)	108	(95.6)	0.92
How long should a TB patient be provided DOTS	10	(90)	25	(92.6)	18	(72.0)	110	(91.7)	108	(95.6)	0.22
What is multidrug resistant TB?	10	(90)	24	(88.9)	13	(52.0)	68	(56.7)	62	(54.9)	0.78
What precautions should a health care worker take when visiting the home or interacting with a TB patient who may be infectious?	10	(90)	17	(63.0)	4	(16.0)	20	(16.7)	33	(29.2)	0.02
**Total knowledge score (%)**	**92**	**(86.30)**	**360**	**(83.3)**	**283**	**(70.8)**	**1424**	**(74.2)**	**1359**	**(75.8)**	**0.27**

*p-value for comparison between tracer and non-tracer facilities.

Values reflect number and percentage correct.

### Attitudes and perceptions

When queried, the reasons most frequently identified for non-adherence to treatment were that patients no longer felt sick (72.1%), personal or cultural beliefs (30.6%), side effects of treatment (30.6%), and fear of stigma (29.8%) (Table 4). Tracer teams were viewed as an effective means to get patients to return to treatment by 96.3% (105/109) of the tracer group respondents. At non-tracer health facilities, lack of human resources was identified as the primary reason for not routinely tracing patients (23/66; 30.3% of respondents). The tracer team leaders also expressed concerns, with 41.7% (10/25) reporting that they did not feel they had sufficient logistical support and 8.3% (2/24) not feeling as though they had adequate supervision and direction from the district or provincial TB managers. Over one-third of team leaders did not feel they had an adequate level of protection or personal safety in areas necessary to travel while tracing TB patients (10/24; 41.7%) and felt inadequately protected from contracting TB from patients (9/25; 39.1%).

**Table 4:** Perceptions by personnel involved in management and treatment of TB on why patients fail to adhere to TB treatment (n = 258)

	**n (%)**
In your experience with TB patients, what is the most common reason that patients do not adhere to treatment?	
Patients no longer feel sick	186 (72.1)
Personal or cultural beliefs	81 (31.4)
Side effects of treatment	79 (30.6)
Fear of stigma	77 (29.8)
Lack of knowledge	63 (27.0)
Lack of access to health care	66 (25.6)
Lack of motivation	47 (18.2)
Feelings of depression or hopelessness about TB disease	37 (14.3)
Poor relationship with health care workers	35 (13.6)
Lack skills to follow medication instructions	28 (10.9)
Language barrier with health care worker	17 (6.6)

### Practices

Almost all (95.5%) respondents from the non-tracer health facilities reported that they routinely conduct activities to track and trace patients who have not adhered to treatment. There were no significant differences between tracer and non-tracer respondents on the timing of tracing activities, with the majority seeking out the patient within 6 days of missing a treatment dose. Tracing activities in both tracer and non-tracer facilities involved home and workplace visits for all patients; there were no significant differences observed between the two groups. A significantly greater proportion of non-tracer health facilities compared to tracer health facilities reported using phone calls to trace patients that missed a treatment dose or a clinic visit (p < 0.001). More than 95% of TB tracer team leaders reported that once the tracer team has contacted a patient who missed an appointment (DOT or sputum appointment), they ask the patient the reason for missing the appointment and they reeducate both the patient and the family on the importance of treatment adherence. Upon returning to treatment at the clinic, tracer facilities were significantly more likely to discuss alternate DOTS arrangements than non-tracer facilities (79.2 vs. 66.4%, p = 0.03).

## Discussion

This study examined the knowledge, attitudes and practices utilized to trace TB patients in South Africa, both among personnel involved with the TB Tracer Project and those that manage TB patients and may conduct tracing as part of standard care within the NTP. This study elucidated specific concerns and aspects that should be considered when integrating tracing activities into routine TB care. Overall, study participants demonstrated a high level of knowledge on core questions regarding the etiology, diagnosis, treatment, and prevention of TB. There was a similar level of overall knowledge between tracer and non-tracer facilities, suggesting that basic TB knowledge did not factor into previously reported differences on the impact of tracing activities [13,14].

The tracer teams were regarded as an effective method for returning patients to treatment, even with the challenges reported by the tracer teams; these findings are similar to previous research [16]. Practices employed by tracer teams focused on returning patients to treatment, patient education and the development of future treatment adherence plans. Non-tracer facilities also reported that they conduct some tracing activities, however, we were not able to assess the frequency of patient home and workplace visits by tracer facilities teams compared to non-tracers. A greater proportion of non-tracer health facilities reported using phone calls to trace patients and non-tracer facilities were more likely to contact the family members of patients than tracer facilities teams. Non-tracer facilities identified lack of human resources as the top reason why they do not routinely trace patients.

While respondents averaged 9.8 years of experience working in TB, it is of concern that just over half of each respondent group (range 50–59.3%) was able to correctly identify the four components of a DOT encounter, namely, verifying the medication, watching the patient take pills, checking for side effects, and documenting the visit. However, the lack of understanding of DOT may be due to only a fraction of respondents reporting having formal training on DOTS (range 53.1-90%). Our findings are similar to those reported from a survey of private practitioners in Ethiopia, wherein only 39.3% had satisfactory knowledge of DOTS [17]. The study also reported that practitioners who had received training on DOTS within the previous 2 years were over four times more likely to have satisfactory knowledge on TB treatment. These findings underscore the critical need to ensure that all personnel involved with TB treatment and monitoring receive comprehensive training on TB care and treatment.

When asked to identify reasons patients fail to adhere to treatment, over two-thirds of respondents cited that patients no longer feel sick, and over a quarter mentioned patient personal or cultural beliefs, side effects of treatment, fear of stigma, lack of knowledge and lack of access to health care. A review of research on patient adherence revealed similar factors leading to non-adherence and default, citing interpretations of illness and wellness as a major factor wherein patients who feel better no longer believe that they are ill and stop taking their medication [16]. Investigators also pointed out that knowledge and attitudes affect adherence, similar to responses expressed by TB personnel in the present study of patient personal and cultural beliefs creating a barrier to adherence. The findings from the current study, in concert with previous research, highlight the need to include patient education emphasizing the importance of completing treatment to effectively cure TB disease as part of standard practice for treating and tracing TB patients.

Activities conducted upon return to the clinic are also important in patient adherence and reuptake of treatment after default. When a patient returns to the clinic, personnel at tracer facilities were more likely to discuss alternative DOTS arrangements with patients than non-tracer facilities. It is possible that these encounters assessed individual barriers to treatment adherence and identified mutually-agreeable methods for providing TB treatment to these patients, which would favor treatment adherence and successful outcomes [10].

Of the tracer team leaders that returned questionnaires, the majority viewed their relationship with health facility staff as very good or excellent, but they also expressed some major concerns. Their concerns included lack of logistical support and supervision and direction from the district or provincial TB managers. They also expressed concerns over their personal safety, both in terms of physical protection in the areas where they were tracing patients and contracting TB from patients. These negative experiences help to identify areas for improvement of future tracing activities in South Africa and other high burden countries.

Some limitations and biases exist in this study. This study was based solely on qualitative and quantitative data from questionnaires. The questionnaires were sent retrospectively with an extended delay from the time of program implementation making this study subject to recall bias. It is possible that some respondents were no longer in the same positions, and those that were may have trouble recollecting activities conducted during the TB Tracer Project. Reported activities may more accurately represent current activities in the program, which may have changed since the project period. However, it is unlikely that the ability to recall information was substantially different between the different groups of respondents. The overall response rate was very low, subjecting our results to bias if respondents were distinctly different than persons that did not complete and return the questionnaires. Our findings may therefore not be representative of the total target population. Our efforts to improve response rates included calling and emailing districts and individuals to remind them to return the questionnaires. Many of the current district staff were not involved at the time of the tracer project and therefore didn’t send feedback and were not included in the study. However, there was little variation in response rates across the provinces that participated in the evaluation. Additionally, it was not possible to evaluate the success among tracer teams to get patients to return to treatment or final patient outcomes, as no information was collected prospectively to track patients identified for tracing individually and the outcome of tracing efforts. The differences revealed in this study between tracer and non-tracer sites are unlikely to have made any substantial contribution to the greater improvements in treatment outcomes at tracer sites compared to non-tracer sites during the TB Tracer Project period [13,14].

## Conclusions

Despite limitations, the current study presents a useful assessment of core TB knowledge among TB program and health facility staff and of practices utilized to increase adherence among TB patients in South Africa. Future studies and health interventions using TB patient tracer teams should include ongoing TB practitioner and tracer trainings. These should stress the need for increased patient education that emphasizes the critical importance of completing treatment irrespective of clinical symptoms. A clear evaluation strategy prior to implementation is also essential in order to accurately understand the relationship of these factors to changes in default and cure rates. Ongoing monitoring and documentation of the frequency of tracing activities will also help to describe differences observed in patient treatment outcomes. These components are vital to identify patient and programmatic factors that may be instrumental in optimizing the success of TB tracing activities.

The study also identified factors that must be considered to optimize the success of tracing activities, including the need for ongoing TB staff education and provisions to ensure physical and personal safety during tracing. The challenges that tracer teams experienced including poor logistical support and fear for safety need to be carefully considered when planning for tracer outreach teams.

## Abbreviations

TB: Tuberculosis; NTP: National TB Program; WHO: World Health Organization; MDR: Multi-drug resistant; DOTS: Directly observed treatment short-course.

## Competing interests

The authors declare that they have no competing interests.

## Authors’ contributions

LJP, LEB, MV, AP and LDM conceived of the study, and participated in its design and coordination. NB participated in the design of the study and follow up to improve response rates. CCB, LJP and LEB developed the analytic plan, and CCB carried out the data cleaning and analysis with support from LJP and LEB. CCB, LJP, and LEB were responsible for the interpretation of results. CCBwas the primary author for writing the manuscript; LJP and LEB also contributed to the writing. All authors read and approved the final manuscript.

## Pre-publication history

The pre-publication history for this paper can be accessed here:

http://www.biomedcentral.com/1471-2458/13/801/prepub
